# A Novel KCNJ11 Mutation Associated with Transient Neonatal Diabetes

**DOI:** 10.4274/jcrpe.5166

**Published:** 2018-05-18

**Authors:** Evangelia Gole, Stavroula Oikonomou, Sian Ellard, Elisa De Franco, Kyriaki Karavanaki

**Affiliations:** 1 University of Athens, 2^nd^ Department of Pediatrics, “P&A Kyriakou” Children’s Hospital, Diabetes and Metabolism Unit, Athens, Greece; 2 University of Exeter Medical School, Institute of Biomedical and Clinical Science, Exeter, United Kingdom

**Keywords:** Neonatal diabetes, KCNJ11, hyperglycemia, transient

## Abstract

Neonatal diabetes mellitus (NDM) is a rare type of monogenic diabetes that presents in the first 6 months of life. Activating mutations in the *KCNJ11* gene encoding for the Kir6.2 subunit of the ATP-sensitive potassium (K_ATP_ ) channel can lead to transient NDM (TNDM) or to permanent NDM (PNDM). A female infant presented on the 22^nd^ day of life with severe hyperglycemia and ketoacidosis (glucose: 907mg/dL, blood gas pH: 6.84, HCO_3_: 6 mmol/L). She was initially managed with intravenous (IV) fluids and IV insulin. Ketoacidosis resolved within 48 hours and she was started on subcutaneous insulin injections with intermediate acting insulin NPH twice daily requiring initially 0.75-1.35 IU/kg/d. Pre-prandial C-peptide levels were 0.51 ng/mL (normal: 1.77-4.68). Insulin requirements were gradually reduced and insulin administration was discontinued at the age of 10 months with subsequent normal glucose and HbA1c levels. C-peptide levels normalized (pre-prandial: 1.6 ng/mL, postprandial: 2 ng/mL). Genetic analysis identified a novel missense mutation (p.Pro254Gln) in the *KCNJ11* gene. We report a novel KCNJ11 mutation in a patient who presented in the first month of life with a phenotype of NDM that subsided at the age of 10 months. It is likely that the novel p.P254Q mutation results in mild impairment of the K_ATP_ channel function leading to TNDM.

## What is already known on this topic?

Neonatal diabetes is a monogenic disorder presenting as a transient or permanent type. Transient cases are usually due to abnormalities in the 6q24 region, while some patients may have mutations in the KCNJ11 and ABCC8 genes.

## What this study adds?

We describe a novel KCNJ11 gene mutation (p.P254Q) in a patient with neonatal diabetes that subsided at the age of 10 months. The p.P254Q mutation seems to cause mild impairment of the ATP-sensitive potassium channel function leading to transient neonatal diabetes.

## Introduction

Neonatal diabetes mellitus (NDM) is a rare form of monogenic diabetes, which usually presents before the age of six months (1). To date, abnormalities in 23 genetic loci have been associated with NDM (2,3,4). Clinically, NDM can be classified into two major categories, transient NDM (TNDM) and permanent NDM (PNDM). The reported incidence of NDM is quite variable and is lower in Western countries [Italy: 1 in 90.000 live births (5), UK: 1 in 400.000 (6)] and higher in Eastern countries [1 in 21.000 in Saudi Arabia (7)], which may be due to high rates of consanguinity. Turkey (particularly its South-Eastern Anatolian regions) has a high rate of consanguineous marriages (40%) and PNDM incidence is reported to be 1 in 48.000 live births (8).

TNDM accounts for approximately 50% of the cases of neonatal diabetes. Children with TNDM are usually born with intrauterine growth retardation (IUGR) and tend to develop diabetes in the first weeks of life (9). Diabetes subsides in the following months, with a possible relapse to a permanent state during puberty or adult life. About 70% of TNDM cases are due to abnormalities in the 6q24 region, with the remainder of patients mainly having mutations in the *KCNJ11* and *ABCC8* genes encoding the Kir6.2 and SUR1 subunits of the pancreatic ATP-sensitive potassium (K_ATP_) channel (10). This channel regulates insulin secretion by linking glucose metabolism and consequent ATP production to calcium-dependent release of insulin. Activating KCNJ11 or ABCC8 mutations lead to inappropriate activation of the K_ATP_ channel, thereby compromising membrane depolarization and insulin secretion (11). Some of these mutations have been reported to have less severe effects on channel function, causing TNDM (12).

In children with PNDM diabetes does not remit, and about half of them have K_ATP_ channel mutations (1). There is significant clinical overlap between the two types of neonatal diabetes and it is therefore not possible to predict the clinical course at the time of diagnosis. Sulfonylureas have inactivating effects on the K_ATP_ channel, hence most of the patients with confirmed KCNJ11 and ABCC8 mutations may discontinue insulin and be successfully managed with oral sulfonylureas (13). In this article, we describe a case of TNDM due to a novel mutation in the *KCNJ11* gene.

## Case Report

A female infant was born to a single mother of Pakistani origin at 38 weeks of gestation. The mother was a refugee and had under her care three healthy children (age: 8, 5 and 2.5 years old), while the presumed father had presented with type 2 diabetes mellitus (T2DM) at the age of 30 years, which also affected many members of his family. The mother had inadequate antenatal care during this pregnancy. Delivery was uneventful and the infant had no dysmorphic features. Birth weight was 2500 g (-2 standard deviations according to World Health Organization growth charts). The patient was admitted to hospital at the age of 22 days, with respiratory distress and signs of severe dehydration. Diagnostic work-up revealed hyperglycemia with severe ketoacidosis (glucose: 907mg/dL, blood gas pH: 6.84, HCO_3_: 6 mmol/L), that was managed with intravenous (IV) fluids and IV insulin administration. In addition, due to the patient’s critical condition, the possibility of infection was also considered and IV antibiotic administration was started, which was discontinued as the results of blood cultures proved to be negative. Hemoglobin A1c (HbA1c) levels on admission were 7.5% (RR:4.0-6.0%). Due to the patient’s young age (less than six months) and the laboratory findings of severe hyperglycemia and ketoacidosis, the diagnosis of NDM was considered. The infant recovered from ketoacidosis within 48 hours and was started on subcutaneous insulin with intermediate acting insulin NPH twice daily, requiring initially 0.75-1.35 IU/kg/d. Blood glucose levels were found to increase significantly after breastfeeding, therefore it was decided to start feeding with specific amounts of expressed breast milk at 150 mL/kg/day. With this regimen, hyperglycemia was well controlled with no episodes of hypoglycemia (blood glucose levels ranging between 91-109 mg/dL). Further investigations did not reveal any signs of autoimmunity with negative anti-glutamic acid decarboxylase (GAD) antibodies (GAD; 0.2 U/mL, RR:<0.9) and antibodies against tyrosine phosphatase-related islet cell antigen 2 (IA-2; <0.1 U/mL, RR<0.75), while cardiologic, ophthalmologic and neurologic examinations revealed normal findings. At the time of diagnosis C-peptide was low (0.51 ng/mL, RR: 1.77-4.68).

**Figure 1:**
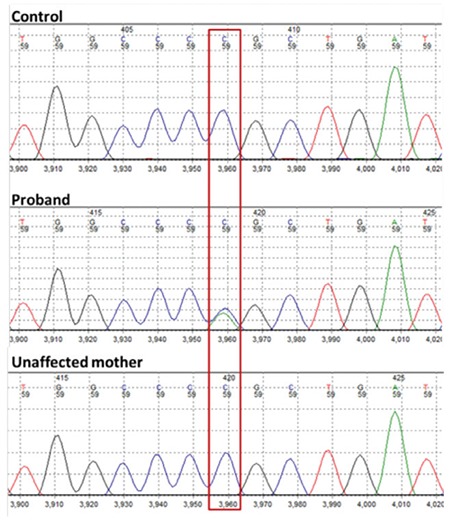
Sanger sequencing analysis of the *KCNJ11* gene. Detection of a novel mutation, c.761C>A (p.P254Q), in the proband

During the patient’s regular follow-up, insulin requirements were gradually reduced and at the age of eight months the patient was requiring 0.32 mg/kg/day of insulin NPH to achieve normoglycemia (HbA1c: 5.4%, RR: 4.0-6.0%). Growth and psychomotor development were normal (weight: 50^th^ percentile, height: 75^th^-90^th^ percentile, head circumference: 25-50^th^ percentile). Informed consent was obtained from the patient’s mother for genetic analysis and publication of the results. Sanger sequencing analysis of the *ABCC8*, *KCNJ11*, *INS* and *EIF2AK3* genes identified a novel missense variant in the *KCNJ11* gene p.Pro254Gln (p.P254Q) (Figure 1). This variant has not been reported before and is not listed in HGMDpro. In addition, the variant has not been identified in 138.487 individuals in the GnomAD database (http://gnomad.broadinstitute.org/). Testing for all the other known neonatal diabetes genes by targeted next generation sequencing (3) gave negative results, confirming that this was the only likely pathogenic variant identified in our patient. *In silico* analysis by SIFT and PolyPhen2 was performed. This analysis predicted that this mutation affects the protein’s function.

The p.P254Q mutation was not detected in the mother’s sample. The presumed father refused genetic testing. Trial of treatment with oral sulfonylureas was planned. However, at the age of 10 months, diabetes remitted and insulin injections were discontinued by the mother, before initiating the scheduled sulfonylurea treatment protocol. Blood glucose levels remained at the normal range without any treatment, HbA1c (4.9%, RR=4.0-6.0%) and C-peptide levels had normalized (pre-prandial: 1.6 ng/mL, postprandial: 2 ng/mL; RR: 1.1-4.4). During the following three months after insulin discontinuation, the patient’s HbA1c (5.2%, RR=4.0-6.0%) and blood glucose levels remained normal.

## Discussion

We report a patient with NDM caused by a novel heterozygous KCNJ11 mutation. Although the p.P254Q mutation has not been reported before, the phenotype of our patient, along with the fact that no mutations were found in the other known NDM genes, supports the pathogenicity of the mutation. This novel mutation (c.761C>A, p.P254Q) leads to the substitution of the non-polar proline at codon 254 for a polar glutamine in the cytoplasmic domain of the K_ATP_ channel. The proline residue at position 254 is highly conserved across species (up to C. Elegans, 23 species considered) and is predicted to be pathogenic by SIFT and PolyPhen2 as described above.

More than 30 activating KCNJ11 mutations have been associated with NDM so far (1). The majority of these mutations seem to affect the K_ATP_ channel’s sensitivity to ATP and impair its function. Mutated K_ATP_ channels show reduced sensitivity to ATP inhibition, resulting in membrane hyperpolarization and impaired insulin secretion (14). Mutations within the ATP-binding site are known to be associated with milder phenotypes, whilst mutations located in areas responsible for channel opening and closure, affect ATP sensitivity indirectly and cause a more severe phenotype (15). The extent of membrane hyperpolarization caused by each mutation can explain the spectrum of variation of the clinical phenotype of the disease, ranging from TNDM (16) to PNDM with neurological complications (developmental delay, epilepsy and neonatal diabetes syndrome) (15). Psychomotor development was normal in our patient.

On the other hand, less severe KCNJ11 mutations result in remitting/relapsing neonatal diabetes, maturity onset diabetes of the young or T2DM at older ages (10). These mutations usually result in mild impairment of K_ATP_ channel function, as has been shown for the p.V252A mutation (17), that is located just two amino acids apart from the mutation identified in our patient. The mechanism proposed to explain these phenotypes suggests that there is a mild beta cell defect caused by some mutations that may be compensated transiently, and that the hyperglycemia may present again in periods of increased insulin requirements (16,18). Although we were not able to perform a functional study, considering the phenotype of our patient, we can hypothesize that the mutation identified in our patient causes a mild reduction in channel sensitivity to ATP. Likewise, our patient’s presumed father developed T2DM at the age of 30 years, although we do not know if he has the same mutation with our patient since he refused genetic analysis.

Managing infants with NDM presents many problems, arising from the very small insulin doses required, the high risk of hypoglycemia, the lack of subcutaneous fat and the coordination of insulin therapy with the frequent and uncontrolled feeding schedule of the newborn period. Continuous subcutaneous insulin infusion (CSII) has been recommended as the treatment of choice in the initial management of infants with NDM (19,20,21). Rapid acting insulin preparations (Lispro, Aspart and Regular) may cause severe hypoglycemia and should be avoided, with the exception of CSII (1), while long acting or intermediate acting insulin has been successfully used in these patients (22,23,24). Due to the low socioeconomic and educational level of the family, our patient was managed with subcutaneous injections of insulin NPH, a treatment which proved to be successful. The patient achieved very good metabolic control with no significant glycemic variability and optimal growth and development. 

Sulfonylurea is the treatment of choice in patients with KCNJ11 or ABCC8 mutations (13,25). Thus after genetic identification of a mutation in one of these genes, more than 400 patients have been successfully transferred from insulin to sulfonylurea and most of them responded well with improved glycemic control and less hypoglycemic events (25). In our patient, the diabetic state remitted before the planned sulfonylurea treatment initiation.

In conclusion, we report the case of an infant with transient neonatal diabetes associated with a novel mutation of the *KCNJII* gene. Diabetes remitted after 10 months with an uneventful course and good psychomotor development under subcutaneous insulin regimen. Studies describing the genotype-phenotype correlation of novel mutations can help clinicians to predict the severity of the disease and appropriately manage these patients.
